# The Prevalence of Alzheimer’s Disease in China: A Systematic Review and Meta-analysis

**Published:** 2018-11

**Authors:** Kanglan LI, Shouchao WEI, Zhou LIU, Li HU, Jiajing LIN, Shiting TAN, Yingren MAI, Wanjuan PENG, Hui MAI, Qi HOU, Guifeng TU

**Affiliations:** 1. Guangdong Key Laboratory of Age-Related Cardiac and Cerebral Diseases, Affiliated Hospital of Guangdong Medical University, Zhanjiang, China; 2. Institute of Neurology, Guangdong Medical University, Zhanjiang, China

**Keywords:** China, Meta-analysis, Alzheimer’s disease, Dementia, Prevalence, Systematic review

## Abstract

**Background:**

Several studies have investigated the prevalence of Alzheimer’s disease (AD) among the general population in several parts of China. However, the results have been inconsistent. This meta-analysis was conducted to estimate the overall prevalence of AD between 2007 and 2017 in China.

**Methods:**

English and Chinese electronic databases were searched with a date range from Nov 2007 to Nov 2017 and the reference lists of the included studies were screened as well. Cross-sectional studies addressing the prevalence of AD among the general Chinese population were retrieved irrespective of the age, location or sex of the participants. Study quality was assessed using the recommended checklist of STROBE.

**Results:**

Overall, 184058 subjects and 7445 patients with AD were included from 17 studies in this meta-analysis. The overall prevalence of AD in China was calculated to be 0.04(95% CI:0.04–0.05). The prevalence was higher in older age groups, among females, and in the rural areas of the country, with an increasing trend in recent years.

**Conclusion:**

AD is a common problem among those in the Chinese population older than 65 yr. Furthermore, an increasing trend of the disease over the past 10 years is indicative of a critical public health problem in China in the near future. Further evidence based on a national survey is needed to estimate the exact prevalence of the disease in the country.

## Introduction

Alzheimer’s disease (AD) is the most common cause of dementia and the most common neurodegenerative disease, affecting millions of predominantly elderly individuals worldwide. In addition, this chronic disease is one of the major contributors to disability and causes increases in the burdens of patients and the families of patients, as well as of the health and social care systems(1). Thus, higher mortality rates and lower life expectancies are seen in AD patients relative to the general Chinese population (2).

The National Aged Population Office has published research in the Future Trends of Population Aging in China, which predicted that, by 2020, 248 million aged people, accounting for 17.17% of the whole population, will reside in China, and that aged 80 yr or above will amount to 30.67 million, occupying a share of 12.37% of the total aged population (3). In particular, unique characteristics of the aging population were seen in China, which has the largest aged population in the world compared with those of developed countries (4).

Despite the presence of one-fifth of the worldwide aging population in China (5), few epidemiological studies of AD have been conducted there in past ten years. The crude prevalence of AD in China has been found to range between 7 per 1000 people to 66 per 1000 individuals (5). In Beijing (northern-eastern), Zhengzhou (northern-central), Guiyang (southern-western) and Guangzhou (southern-eastern), the prevalence of AD was 3.21% in a total of 10276 residents aged 65 yr or older, the prevalence in China was lower than that of the developed western countries(6,7). WHO report on dementia adopted the estimates of Alzheimer’s Disease International but acknowledged their limitations and called for further epidemiological studies in low-income and middle-income countries (8). Whether the prevalence of AD in China was previously underestimated is unknown. In this study, we aimed to estimate the prevalence of AD based on all of the available published studies, resulting in the first meta-analysis of the prevalence of AD in China between 2007 and 2017.

The aims of the present study were as follows: to determine the prevalence of AD over the past ten years; and to identify the gender, location and age distributions of the patients. This review on the prevalence of AD could not only improve the social focus on the needs of seniors but could also guide us in preventing the disease. China’s academic databases are a valuable and largely unexplored resource for understanding the epidemiology of AD. We also investigated the differences in prevalence by age and gender and between rural and urban regions in order to inform policy and priority setting.

## Methods

### Search Strategy

To obtain Chinese epidemiological data, we searched PubMed, EMBASE, the Chinese National Knowledge Infrastructure database (CNKI), the Chinese Biological Medical Literature database (CBM), and the Chinese Wanfang and Chongqing VIP database for studies using the free combinations of the terms “Alzheimer’s disease,” “Prevalence,” and “China” in both the English and Chinese languages. The search was limited to studies published between Jan 1, 2007, and Nov 30, 2017. The reference lists and reviews were also assessed to find any additional studies considered relevant to the topic.

The authors of the included studies were contacted as well. Two independent reviewers (Shouchao Wei and Kanglan Li) studied all of the identified titles and abstracts independently. In addition, they reviewed the full texts to extract studies that met the inclusion criteria. Any disagreements were resolved by adjudication with a third author (Zhou Liu). The variables extracted for the data analysis included each study design, the year and location of each study, sample size, number of outcomes, mean age of participants, and gender of the participants.

We used the evaluation criteria for *strengthening the reporting of observational studies in epidemiology* (9) to evaluate the included studies. The evaluation criteria included (A)–(F). Satisfied all criteria were considered to be of high quality. Intermediate-quality did not fulfil one criterion. Low-quality did not meet more than one criterion.

(A) The outcomes were defined clearly.
(B) The criteria were appropriate.
(C) The study included the key points.
(D) The number of outcome events was reported.
(E)The subjects were recruited in a clear way.
(F) The locations, dates, setting were described.

We excluded studies of duplicate population groups that were of lower quality, those that had participants drawn from a particular occupation or population, and those that did not satisfy one or more of the inclusion criteria.

### Inclusion Criteria

Studies with any of the following characteristics were included:

(A) Authentic population-based epidemiological studies, written either in English or in Chinese that were able to provide sufficient information to estimate the prevalence of AD;
(B) Based on general population samples rather than on a special population;
(C) The study population was scrutinized with validated diagnostic criteria, with accurate dates included;

### Exclusion Criteria

Studies with any of following characteristics were excluded:

(A) Reviews, editorials, letters, commentaries, and reports;
(B) Studies from which the data had been already included by another study;
(C) Studies of duplicate population groups that were of lower quality, such as those having unclear results, or those that did not report relevant data;
(D) The participants were recruited from a particular occupation population or other population and those that did not satisfy one or more inclusion criteria.

### Data Extraction and Quality Assessment

Two authors (Shiting Tan and Shouchao Wei) independently extracted data from each study, and any disagreements were discussed. The information pertaining to the study were extracted and entered into tables as follows: name of the first author, date of investigation, location, study region, sample size, age of participants, age group, diagnostic criteria, and prevalence of AD.

### Statistical Analysis

The Review Manager 5.3 (Review Manager, Copenhagen: The Nordic Cochrane Center, The Cochrane Collaboration, 2010) statistical software package and SPSS 17.0 (Chicago, IL, USA) were used for the data analysis.

In our present study, the chi-square test was used to test the statistical heterogeneity of the data at the 5% significance level. I^2^ statistic and tau-square were adopted to quantify the inconsistency and variance of the study’s results, respectively (10). To assess publication bias quantitatively, Begg (11) and Egger (12) statistical tests were used.

A meta-analysis was performed to obtain a summary measure of the “prevalence” of Alzheimer’s disease in the general population. Data were analyzed, and random-effects model was used to the report of prevalence with 95% confidence interval (CI). Pooled estimates were calculated using log-transformed proportions. Subgroups were defined by gender for subgroup analyses; age groups were also created to account for heterogeneity and to perform secondary analyses.

## Results

**Fig. 1:**
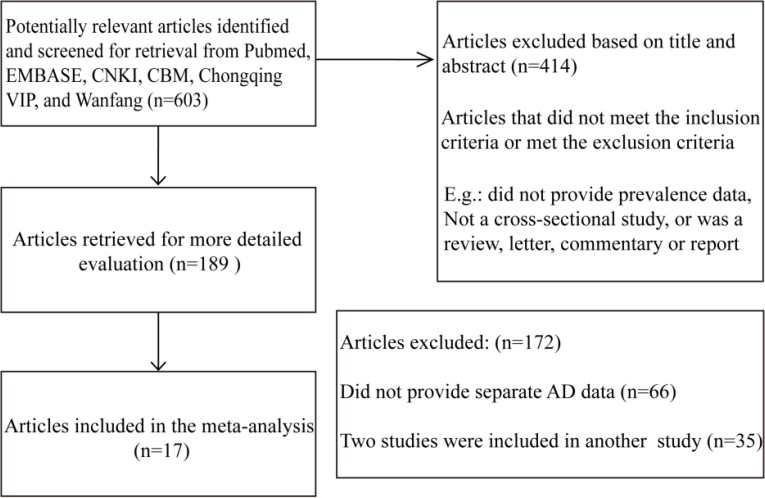
Flow diagram of the study identification process

The process used to identify the eligible epidemiological studies is summarized in Fig. 1. By combining both the criteria outlined in the methods section and reviewing the lists of titles and abstracts, we identified 17 full-text articles from all of the databases for further review. The PubMed and EMBASE searches yielded 4 potentially relevant articles, and the Chinese database searches yielded 13 articles. Studies of nervous system diseases that did not provide separate AD data or that were irrelevant (n= 15) and studies included in an already published study were excluded. Seven studies were of special populations (e.g. groups from hospitals and the army) were excluded. Therefore, 17 unique studies were included in our study based on the full-text reviews (6, 13–27).

### Description of studies

The characteristics of all studies in the review are shown in Table 1. Overall, 184058 subjects and 7445 patients with AD were included from the 17 studies in this meta-analysis. All of the included studies were conducted between 2007 and 2017 and covered 7 provinces and 8 municipalities in China, including Beijing, Shanghai, Guangzhou, Guiyang, Zhengzhou, Ningbo, Tianjin, Fuzhou, Jiangxi, Zhejiang, Hainan, Hebei, Gansu, Shanxi and Inner Mongolia.

**Table 1:** The characteristics of all studies in the review

***First author and year published***	***Survey***	***Level (National/Provincial/Municipal)***	***Location***	***Geographical Area (latitude-longitude)***	***Rural/Urban***	***Age range***	***Diagnostic criteria***	***Total sample size***	***Total case size***	***Prevalence***
Li H et al.(2009)	2007	Municipal	Fuzhou	Southern-Eastern	Rural	≥65	DSM—IV	2696	131	5%
Huriletemuer et al.(2011)	2008–2009	Provincial	Inner Mongolia	Northern-Central	Urban	≥55	NINCDS-ADRDA	9266	448	4.79%
YE et al.(2011)	2009	Municipal	Ningbo	Southern-Eastern	Urban	>60	BSSD DSM-IV-R	2445	135	5.53%
Kang et al. (2011)	2012	Provincial	Hebei	Northern-Eastern		≥60	DSM-IV	3632	177	4.87%
Lao et al. (2011)	2010	Provincial	Hainan	Southern-Eastern		≥55	HDS CDR	7665	111	1.45%
Fan et al. (2011)	2008	Provincial	Shanxi	Northern-Western		≥60	DSM—IV	1826	38	3.80
Sun et al. (2012)	2010–2011	Municipal	Shanghai	Southern-Eastern	Urban	≥60	NIA-AA criteria	1472	56	3.8
Ding et al. (2013)	2012	Municipal	Shanghai	Southern-Eastern	Urban	≥60	DSM-IV; NINCDS-ADARDA	3141	113	3.6
Ma et al. (2013)	2010	Municipal	Shanghai	Southern-Eastern		≥65	ICD-10	2442	101	8.44
Chen et al. (2013)	2010	Provincial	Jiangxi	Southern-Central		≥60	NIA-AA criteria	1554	89	5.72
Ding et al.(2013)	2012	Municipal	Shanghai	Southern-Eastern	Urban	>60	NINCDS-ADRDA	3141	113	3.6
Jia et al. (2014)	2014	National	Beiing, Guangzhou, Zhengzhou, Guiyang	ALL	Rural/Urban	≥65	NINCDS-ADRDA	10276	330	3.21
Zou et al. (2014)	2012–2013	Municipal	Haining city	Northern-Eastern		>60	NINCDS-ADRDA	121949	4795	5.61
Li C et al. (2015)	2014	Municipal	Tianjin	Northern-Eastern		≥60	NINDS—ADRDA	2532	144	5.69
Ji et al.(2015)	2011–2012	National	North China	Northern	Urban	>60	DSM-IV	5537	299	5.4
Li et al. (2016)	2014	Provincial	Zhejiang	Southern-Eastern		>60	NIA-AA criteria	2015	239	6.9
Zhang et al.(2017)	2015–2016	Municipal	Gansu	Northern-Western		≥55	CDRH I S	2242	64	2.85

The maximum sample size of the study was 121949 from a municipal survey in 2012, and the minimum was 1472 from a study conducted in Shanghai. All of the studies were conducted using door-to-door surveys over two phases: an interview screening followed by the confirmation of a positive result of the screening. All of the included studies were high-quality.

Among the included studies, the participants’ age of onset was 55 and above in three studies, 65 and above in four studies, and 60 and above in the remaining studies.

In most of the studies, the diagnostic criteria of AD were based on the presence of at least two of the four cardinal signs and the exclusion of other diseases. The target population in two studies lived in urban areas, and two did not refer to the location, while the rest of the studies had recruited samples from both urban and rural areas. These studies reported an AD prevalence ranging from 183 to 844 per 10000.

### Estimated prevalence of AD

The combined result for the pooled prevalence was 0.04 (95% CI: 0.04–0.05) based on the random effects model over the recent 10 years, while the maximum and minimum prevalence in AD were observed in Shanghai (8.44%) and Hainan (1.41%), respectively (Table 1).

**Fig. 2:**
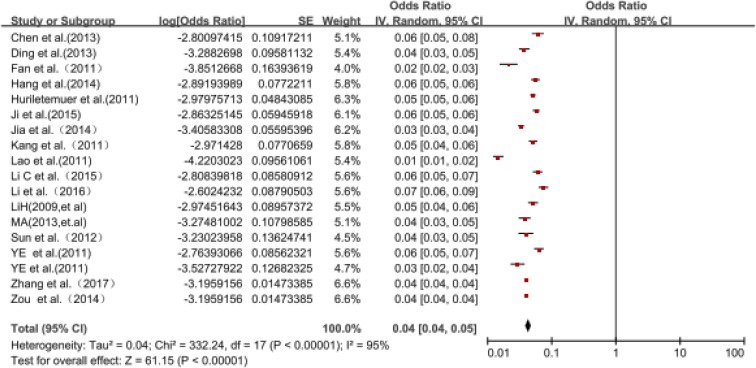
Forest plot for the pooled prevalence and confidence intervals of AD in China obtained from the meta-analysis (arranged by survey chronologically from 2007 to 2017)

The prevalence of AD was estimated according to years and the pooled prevalence estimates varied by year (Table 2, Fig. 2). The prevalence of AD increased significantly from 404 per 100,000 people in 2007 to 624 per 10,000 people in 2014.

**Table 2:** The studies of the prevalence of AD stratified by age group and gender by years

***Trends in the prevalence of AD by gender and age: 2007–2017***									
***Survey year***	***2007***	***2008***	***2009***	***2010***	***2011***	***2012***	***2013***	***2014***	***2015***
Overall prevalence (%)	4.4								
Prevalence by year (%)	3.44	3.82	5.53	4.63	5.42	4.92	5.26	8.42	2.85
Gender (%)									
Male	3.34	3.22	3.64	3.98	3.77	4.35		8.82	2.01
Female	6.23	9.84	7.21	5.94	6.74	5.84		14.1	3.65
Age(yr) (%)									
55–59	/	/	/	0.35	/	/	/	/	/
60–64	/	/	1.21	1.47	2.74	/	/	2.27	/
65–69	1.11	1.58	2.45	1.63	1.76	/	1.83	8.51	/
70–74	1.74	3.53	3.55	3.02	4.1	/	8.98	12.92	/
75–79	7.92	7.85	5.92	10.81	8.65	/	9.95	18.38	/
≥80	33.5	23.64	17.85	22.67	29.82	/	/	26.77	/

There were two different age-group ranges used in the studies: one included the age ranges of 55–64, 65–74, 75–84 and 85+ yr and was used by two studies, and the other included the age ranges of 50–59, 60–69, 70–79 and 80+ yr and was used in six studies. Nine studies did not provide information for the AD patients according to gender

### Subgroup analysis

We stratified the included studies based on location and gender for prevalence.

### Prevalence by location-specific

**Fig. 3:**
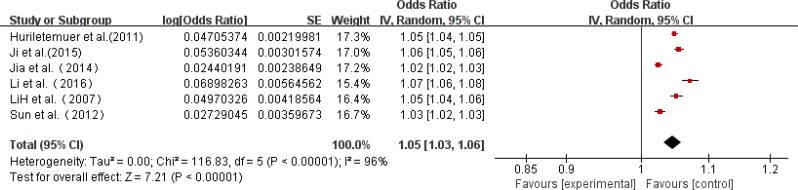
A forest plot displaying the pooled prevalence of AD in rural regions in China

**Fig. 4:**
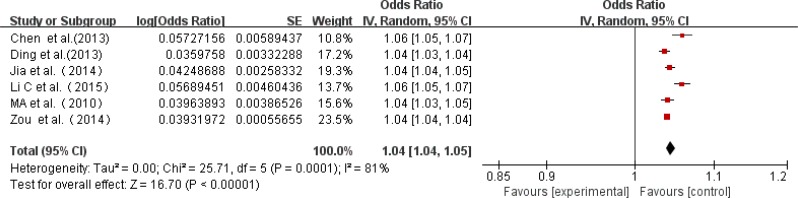
A forest plot displaying the pooled prevalence of AD in urban regions in China

Six studies reported prevalence data for rural and urban respectively. Among the 12 studies, which included 1166 rural patients and 5501 urban patients, the rate of AD ranged from 344 to 689 per 10,000 rural patients, and 359 to 572 per 10,000 urban patients. A higher prevalence of AD was found in rural than urban (95% CI 4.27–4.78 *vs* 3.89–4.09) and the prevalence was 452 per 10,000 and 399 per 10,000 for rural and urban patients, respectively (Table 3, Figs. 3 and 4).

**Table 3:** Overall and mean prevalence of AD by locations and gender

***Items***	***Number***	***Case***	***Population***	***Prevalence (%)***	***95%Confidence intervals(CI)***
Locations					
Rural	6	1166	25787	4.52	4.27–4.78
Urban	6	5501	137879	3.99	3.89–4.09
Gender					
Male	12	623	20696	3.01	2.78–3.24
Female	12	1459	24789	5.89	5.59–6.18
Overall	18	7345	184058	3.99	3.90–4.08

### Prevalence by gender-specific

**Fig. 5:**
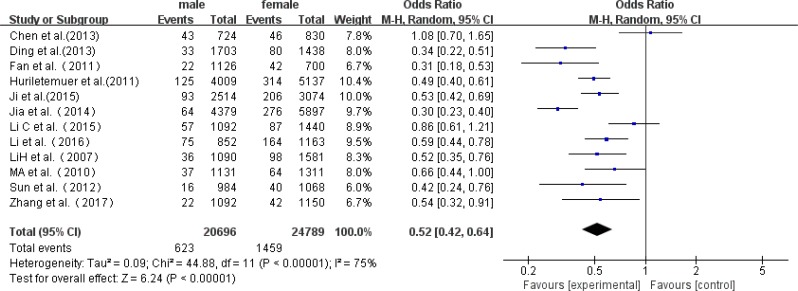
A forest plot displaying the risk of AD among females *vs*. males in China

Twelve studies reported prevalence data for both genders, while six studies only investigated male or female patients. Among the 12 studies, which included 623 affected males and 1459 affected females, the rate of AD ranged from 162 to 594 per 10,000 males, and 365 to 670 per 10,000 females. A higher prevalence of AD was found in females than in males (95% CI 2.78–3.24 *vs* 5.59–6.18), and the prevalence was 301 per 10,000 and 588 per 10,000 for males and females, respectively (Fig. 5, Table 3).

### Heterogeneity and publication bias

There was considerable heterogeneity among the included studies, such that the result of the Chi^2^ test for heterogeneity was highly significant (*P*<0.001). In addition, the I^2^ statistic was calculated to be 97%. To reduce the heterogeneity, we divided the studies into subgroups according to gender and location to achieve homogeneity. Nonetheless, homogeneity was not achieved. The results of the statistical tests for publication bias, including the Begg and Egger tests, confirmed the presence of publication bias.

## Discussion

With increasing life expectancy, AD is becoming a noticeable public health problem worldwide, particularly in the largest developing country, China. Our study is the first one of AD in mainland China between 2007 and 2017.

This meta-analysis showed the results of several independent cross-sectional studies conducted in the Chinese mainland, which suggested that the overall prevalence of AD was lower than that of some developed countries (28). The main reason for this can be owing to the shortage of methodological uniformity. The knowledge and attitudes toward AD have also changed over the years, affected diagnostic standards and measurement methods. Clinicians have different interpretations of clinical diagnostic criteria and the resulting bias is hard to avoid. In addition, the sample size and the limitation of the case sources are also important reasons the prevalence rate of AD dropped sharply in 2015.

Examination of the age-specific prevalence of AD presented an increase with age. The prevalence of AD increases with age and shows gender differences. The prevalence of AD varies in different populations, age groups, locations (rural or urban) and areas. The prevalence of AD varies greatly worldwide and that the standardized prevalence (all ages) ranged from 207 to 812 per 10000 according to door-to-door surveys (29). The overall prevalence of AD was lower in Western countries, such as the USA (30) and Britain (31). Comparing the current prevalence of AD in Asian countries, South Korea was found to have the prevalence of AD increased from 5.0% to 6.5% in the past two decades (32,33).

The variations in the AD prevalence across the different studies might provide clues regarding the influence of different environmental exposures, susceptibility genes and so on. AD is most commonly sporadic, but the familial type accounts for approximately 5% of AD patients and is considered to be due to the combination of genetic and environmental factors (34). In India, population structure and socioeconomic status might be associated with the low prevalence (35). The lack of biomarkers and the dependence on clinical evidence are two concerns regarding the diagnosis of AD, and undertaking a neurological examination, especially in very elderly people, is often difficult.

As AD is mainly an illness later in life, it is more common in developed countries, where the prevalence is higher. However, the trend towards a lower prevalence in less-developed regions than in developed settings has been endorsed, at least for sub-Saharan Africa and South Asia (36). The quality and coverage of the evidence base are poor, with very few published studies from Latin America, Africa, the Middle East, Eastern Europe, and Russia, and patchy and inconsistent estimates in other less developed regions (37). Different regions of the Chinese mainland are developing differently. The Changjiang Delta region (e.g. Shanghai and Zhejiang Province) is the most economically developed area in Chinese mainland; the urban-rural integrated health care system there has a demonstration effect on a nationwide scale.

Hainan, as the only tropical island in China, enjoys the lowest prevalence rate. Inner Mongolia is the least developed western region in China, as Mongolian is the main ethnic minority autonomous area. There is a rich diet culture and lifestyle, especially in regard to the Mongolian cuisine, and the dining traditions are emblematic of the local culture. Compared with the Mongolian groups, those in the Inner Mongolia area, who are of the Han groups, have significant differences in their genetic characteristics.

AD is a progressive, disabling neurodegenerative disorder characterized by senile plaques and neurofibrillary tangles (38). In addition, variations may be caused by the population structure, as well as by genetic and environmental factors. Moreover, possible explanations for the variation in prevalence include population characteristics and the different diagnostic criteria used in different areas. There is a close relationship between AD and environmental exposures. The reasons for the prevalence differences among the studies may be due to etiology, including different susceptibility genes or different environmental exposures between the studies. Cigarette smoking, the consumption of ethyl alcohol, aluminum and exposure to environmental heavy metals have all been reported as risk factors for developing AD from compelling epidemiological data (39,40).

However, China is also the largest smoking and drinking country in the world; in addition, the number of individuals living is large, and there are more opportunities for exposure to nicotine and ethyl alcohol. The reported differences may, therefore, have been the result of individuals having more opportunities for exposure.

Increasing age is the most significant risk factor for AD (41). The accumulation of mitochondrial DNA deletions during aging causes age-related genetic changes in the tau protein and amyloid beta. The functioning of neurons is affected in AD by such hyperphosphorylated tau and by amyloid beta accumulation (42, 43). Furthermore, one of the major contributors to the pathogenesis of AD is oxidative stress via the generation of free radicals, which plays a crucial role in aging. The results of this meta-analysis showed that the prevalence of AD clearly increases with increasing age and steeply increases after the age of 65, similarly seen for most neurodegenerative diseases (44). Globally, the prevalence of AD increases exponentially with age, approximately rising from 3% among that 65–74 yr old, to almost 50% among those 85 yr old or older (45). In particular, the number of older adults (aged 65 yr and older) is projected to increase even more dramatically, and 5% of Europe’s individuals above the age of 65 are affected by the disease.

Generally, the prevalence of AD seems to be higher in females than in males, and several studies (46) have confirmed this finding. Possible reasons for this discrepancy between males and females include the neuroprotection offered by estrogen; however, female estrogen levels, which are most abundant before menopause, have shown no consistent association with AD (47). Perhaps the estradiol level before menopause affects processes that later lead to AD. Testosterone is much more abundant in men than in women (48), and testosterone levels are positively associated with cognitive function in both elderly men and elderly women, whereas endogenous estradiol levels were either not associated or were negatively associated with cognition(49,50).

There were several limitations and potential publication biases in this meta-analysis. First, the different diagnostic criteria used may have been a potential source of the variation among the studies. The diagnosis of AD is based on clinical criteria, which can have different standards. Second, an unavoidable factor was the different levels of training and clinical experience of the investigators. Additionally, it is possible that the patient assessments used in a single period may not have been accurate enough for the studies. Generally, diagnosis is sometimes difficult and uncertain during the early stages of AD.

Sometimes, it is necessary to observe responses to disease progression and to therapy for a long time. Treatment effects may have impacted the disease prevalence, as well as a lack of data regarding the treatment of AD. These studies were excluded from this meta-analysis as this issue might have introduced selection bias into our results. Another factor is the differences among the recruitment methods. Some studies recruited untreated, younger onset cases or retirement organization cases, as this resulted in a larger sample size.

In addition, the calendar time of the included studies was a very broad range and, therefore, the applicable standardized and adjusted crude rates of AD could not be provided. The diagnosis of AD needs to be further assessed, as only long-term follow-ups can be used to confirm the diagnosis.

## Conclusion

This meta-analysis summarized the prevalence of AD in China. There were significant differences in the prevalence in terms of location and gender. Additionally, the prevalence of AD increased with age, with the highest rate seen in the groups above 80 yr old. A few studies have provided data for different stages of the disease. In addition, because of the considerable heterogeneity among the results of the studies, further evidence based on a national survey is needed to estimate the exact prevalence of AD in the country.

## Ethical considerations

Ethical issues (Including plagiarism, informed consent, misconduct, data fabrication and/or falsification, double publication and/or submission, redundancy, etc.) have been completely observed by the authors.
